# Placental Maternal Vascular Malperfusion is Associated with Prepregnancy and Early Pregnancy Maternal Cardiovascular and Thrombotic Profiles

**DOI:** 10.3390/reprodmed3010006

**Published:** 2022-03-08

**Authors:** Carole A McBride, Ira M Bernstein, Amelia B Sybenga, Kelley C McLean, Thomas Orfeo, Maria Cristina Bravo

**Affiliations:** 1 Department of Obstetrics, Gynecology & Reproductive Sciences, University of Vermont Larner College of Medicine, Burlington, VT, United States.; 2 Department of Obstetrics, Gynecology & Reproductive Sciences, University of Vermont Larner College of Medicine, Burlington, VT, United States.; 3 Department of Pathology, University of Vermont Larner College of Medicine, Burlington, VT, United States.; 4 Department of Obstetrics, Gynecology & Reproductive Sciences, University of Vermont Larner College of Medicine, Burlington, VT, United States.; 5 Department of Biochemistry, University of Vermont Larner College of Medicine, Burlington, VT, United States.; 6 Department of Biochemistry, University of Vermont Larner College of Medicine, Burlington, VT, United States.

**Keywords:** malperfusion 1, placenta 2, preeclampsia 3, pregnancy adaptation 4, thrombin generation 5

## Abstract

Characteristics of maternal vascular malperfusion (MVM) are frequently observed in placentas from pregnancies impacted by preeclampsia, intrauterine growth restriction, preterm labor, and intrauterine fetal demise. We sought to evaluate the associations of features of MVM with subclinical measures of cardiovascular health and coagulation potential in healthy young women. Sixty-three healthy young women were recruited and assessed prior to pregnancy on cycle day 9 ± 4, at gestational age 90 ± 6 of early pregnancy, and gestational age 216 ± 5 of late pregnancy. Women were assessed for plasma volume, blood pressure, response to volume loading, cardiac output, and uterine hemodynamics. Platelet-poor plasma was collected to assess thrombin generation on a subset of 33 women at all time points. Following delivery, placentas were collected and analyzed for evidence of MVM. Thrombin generation (TG) was evaluated in the presence of tissue factor (TF) with and without recombinant soluble thrombomodulin (TM). For each, we compared TG lagtime, peak level, and endogenous thrombin potential (ETP). Comparisons were made between dichotomized presence and absence of each individual feature of MVM and cardiovascular and coagulation features. Mean ± standard deviation are presented. Women were 31 ± 4 years of age, body mass index of 24 ± 5 kg/m^2^, 86% white race, and 80% nulliparous. MVM occurred in 70% of placentas, with infarcts and agglutination (44%), decidual arteriopathy (40%), accelerated villous maturation (32%), placental hypoplasia (29%), and distal villous hypoplasia (17%) documented. Decidual arteriopathy and distal villous hypoplasia were associated with prepregnancy maternal physiology, including decreased plasma volume and subclinical cardiovascular variations. All assessed MVM characteristics had identifiable early pregnancy physiologic characteristics consistent with altered cardiovascular function and decreased uterine response to pregnancy when compared with women who did and did not develop MVM. Accelerated villous maturation was the only MVM feature to differ by thrombin generation parameters in early pregnancy. Thrombin generation potential and blood pressure were elevated in late pregnancy in women who developed decidual arteriopathy. Prepregnancy health status and adaptation to pregnancy play important roles in pregnancy outcomes. Both cardiovascular health and thrombin generation potential may influence early placentation. Longitudinal assessment of subclinical maternal factors may allow for better understanding of the etiologies of MVM lesions, as well as allow for identification of a timeline of the origins of placental pathologies.

## 1. Introduction

Disorders of placentation are associated with adverse obstetrical outcomes. These include preeclampsia, gestational hypertension, intrauterine growth restriction, and fetal demise [1–6]. In pregnancies complicated by preeclampsia, placental lesions associated with maternal vascular malperfusion (MVM) are increased and occur more frequently in early onset preeclampsia compared to late onset [7]. Despite this association with poor pregnancy outcomes, as many as 10–50% of pregnancies with healthy outcomes also have evidence of MVM[8], making the overall identification of MVM lesions weakly associated with disease or risk. The term MVM is a blanket term for conditions which influence the exchange of nutrients across the maternal-fetal interface, and includes underperfusion, maladaptive perfusion, as well as high-velocity malperfusion, all of which have the ability to impair normal placental development [1]. The Amsterdam criteria were developed in order to provide greater discrimination of lesion types. These criteria set predefined protocols for histopathological sampling, placental gross descriptors, terminologies, and diagnostic criteria for features of MVM, fetal vascular malperfusion, and characterizations of other placental abnormalities [6]. The process of early placentation, including trophoblast invasion and spiral artery remodeling, are responsible for placental development and are believed to be the primary influence on the development of placental lesions [9, 10].

Maternal prepregnancy health status plays an important role in pregnancy outcomes. Our lab has studied how prepregnancy cardiovascular health and adaptation to pregnancy impact pregnancy outcomes, particularly in the development of subsequent preeclampsia [11–14]. Similar studies have identified the importance of prepregnancy cardiovascular health on placental health, including the occurrence of MVM [15]. Unique vascular adaptations to pregnancy, including how prepregnancy physiology influences adaptation to pregnancy, may play a role in the development of MVM. In support of this, abnormal placental development, particularly in the context of MVM, may be related to future risk of cardiovascular disease in these women [16].

Thrombotic balance may also be a critical contributor to early placentation. Early placentation is a time of abundant micro-clot formation and lysis as trophoblastic invasion sets the stage for the maternal/fetal interface. Overall pregnancy is a prothrombotic condition associated with increased risk of venous thromboembolism and thrombotic stroke. Women who develop preeclampsia have further accelerated thrombotic risk [17, 18]. Additionally, organ damage occurring in preeclampsia has been linked in part to excess thrombin generation [17].

In this study, we sought to explore the relationships between key placental features of MVM with both prepregnancy maternal status and maternal adaption to pregnancy, with particular focus on changes within specific cardiovascular metrics in thrombin generation potential. We hypothesized that women with an impaired cardiovascular response to pregnancy would be more likely to develop features of MVM independent of the development of preeclampsia, and similarly that features of MVM would be identified in women with higher pro-thrombotic potential.

## 2. Materials and Methods

This study was approved by the University of Vermont Institutional Review Board and the separate University of Vermont General Clinical Research Center Scientific Advisory Committee. Written consent was obtained from all participants prior to enrollment.

Sixty-three young, healthy women were recruited through open advertisement prior to planned pregnancy. Participants had no history of hypertension, diabetes, autoimmune disease, or other known chronic medical conditions. Thirteen had a history of preterm preeclampsia but were not hypertensive at the time of assessment. Participants did not use hormonal contraceptives or regular anti-inflammatories, were of BMI 18–40 kg/m^2^, and were predominantly Caucasian.

Study visits occurred in the University of Vermont Clinical Research Center from November 2011 to May 2016. Women were evaluated prior to pregnancy in the follicular phase of the menstrual cycle, and during weeks 12–14 and 30–32 of pregnancy. All study evaluations followed 3-days of a sodium/potassium balanced diet in order to minimize dietary-related hemodynamic variation. Meals were prepared by a registered dietitian and provided to subjects in advance of study visits. Participants abstained from anti-inflammatory medications and decongestants for 48 hours prior to visits, and alcohol and caffeine for 24 hours prior to evaluation. Studies were performed in the post-absorptive state, following an overnight fast. Body composition was measured using DEXA prior to pregnancy.

### 2.1. Physiologic Measures:

Physiologic evaluations occurred after 30 minutes of supine rest, while participants remained supine. Plasma volume was assessed using the Evans Blue Dye method, as previously described [12]. Cardiac output was evaluated by dual Doppler echocardiographic examination [19]. Cardiac ejection time and aorto-femoral velocity were evaluated using dual Doppler ultrasound and EKG, using time from EKG R wave to peak systolic flow in the aortic root [20]. Similarly, pulse wave velocities (PWV) were calculated from EKG R wave to peak systolic flow in the popliteal and brachial arteries and corrected for height and cardiac ejection time. Beta Stiffness and distensibility were calculated from cine clips obtained during Doppler ultrasound from the popliteal artery [21].

Uterine blood flow was estimated using color Doppler ultrasound with an 8.0 MHz transvaginal transducer employing a Vivid 7 General Electric ultrasound unit (Milwaukee, WI). Uterine artery measurements were obtained lateral to the cervix at the level of the internal os, and vessel diameter was measured during real-time color Doppler imaging [12]. Uterine index was calculated as the percent of total blood flow directed to the uterus, in order to evaluate regional redistribution of blood flow associated with pregnancy adaptation.

The Finopres Pro (Enschede, Netherlands), a tonometric blood pressure monitoring system, was used to determine beat-to-beat changes in systolic and diastolic blood pressure, pulse pressure, and mean arterial pressure. These measures were taken both as a baseline, and during a volume challenge prior to pregnancy. During the volume challenge, women were evaluated for vascular compliance, in which a 500 mL bolus of Lactated Ringer’s saline was given over approximately a 10-minute infusion time, and for 15 minutes post-infusion. During this time, women were monitored for changes in blood pressure and pulse [20].

### 2.2. Thrombin generation and biomarkers:

Plasma samples from a subset of 33 women were assessed using thrombin generation assays. At each study visit, blood was drawn and processed to platelet-poor citrated plasma, EDTA plasma, or serum and stored at −80^o^C for later batched analyses. Thrombin generation (TG) of the citrated platelet-poor plasma aliquots was measured on the Throminoscope (Diagnositca Stao S.A.S., France) using the standardized Calibrated Automated Thrombogram protocol [22]. Briefly, samples were thawed in the presence of corn trypsin inhibitor to prevent contact pathway activation, tissue factor (TF) was added, a subset of samples were assayed in the presence of 10 nM recombinant soluble thrombomodulin (TM, TF+TM), and reactions initiated with the addition of calcium and fluorescent thrombin substrate. Thrombin generation was monitored over time using the Throminoscope software and parameters of TG lagtime, peak level, maximum thrombin generation rate, and endogenous thrombin potential (ETP) were recorded for each sample and condition analyzed in duplicate. Comparisons were made between prepregnancy, first trimester, and late pregnancy within each parameter in the context of individual dichotomized (y/n) MVM lesions. Mean ± standard deviation are presented.

### 2.3. Placental examination:

Medical records were reviewed for pregnancy outcome data. Following delivery, placentas were collected and analyzed for evidence of individual lesions indicative of MVM according to the Amsterdam Criteria [6] and the American Registry for Pathology guidelines[23], by a board-certified pathologist, subspecialized in perinatal pathology and blinded to participant data and pregnancy outcome. Placentas meeting criteria for decidual arteriopathy (DA) required at least three features of DA to be considered affected[23]. Placental weight percentiles were calculated [24], and placental hypoplasia was dichotomized according to greater than or less than the 10^th^ percentile according to placental growth curves [24, 25]. Multiple features of MVM were assessed using a scoring system composed of accelerated villous maturation (AVM), placental infarct and/or villous agglutination (INFAGG), distal villous hypoplasia (DVH), or placental hypoplasia. The presence of 2 or more individual MVM lesion types was dichotomized for prediction through logistic regression analyses [9].

### 2.4. Statistical Analyses:

Each MVM lesion type was dichotomously categorized as either present or absent based on placental examination. Response to volume infusion was analyzed by area under the curve (AUC) for cardiac output, pulse pressure, mean arterial pressure, and pulse, and compared with placental pathology. Baseline characteristics, physiologic measures, and thrombin generation parameters were compared between groups using Students t-tests or Mann-Whitney for continuous variables and Chi-squared tests for categorical variables. Spearman’s correlation coefficients were calculated for continuous variables. Logistic regression was used to determine factors of predictive significance for placentas with clinically recognized MVM, requiring two or more features of MVM. History of preeclampsia was included in all models. Statistical significance was determined based on p< 0.05, and all statistical analyses were performed using SAS statistical software version 9.4 (SAS Institute, Cary, NC).

## 3. Results

Participating women were young and healthy, mean age 31 ± 4 years and BMI 24 ± 5 kg/m^2^. Study population demographics and pregnancy outcomes are presented in Table 1.

Women were healthy at the time of all assessments. Body composition, cardiovascular measures, and uterine hemodynamic measures from the three study visits are outlined in Table 2.

### 3.1. Pregnancy Outcomes

Placental evidence of MVM lesions was very common, with 70% of placentas having at least one assessed MVM lesion. Nineteen placentas had 1 MVM lesion, 11 met criteria for two distinct categories of MVM lesions, 13 had 3 characteristics, and 1 met criteria for evidence of all four assessed features of MVM. There were no significant differences in rates of any MVM lesion by development of preeclampsia in the pregnancy from which the placentas were collected. Of interest, infarcts and villous agglutination INFAGG occurred more frequently in women with a history of preeclampsia. Of the 9 pregnancies complicated by preeclampsia in our study, four were preterm and five were term. Of these, all preterm placentas (n=4) showed evidence of multiple features of MVM, 75% (3 out of 4) having evidence of 3 MVM lesion types. Of term preeclampsia, 60% (3 out of 5) showed evidence of MVM, with 2 of 5 having evidence of 2 forms of MVM lesions.

### 3.2. Decidual arteriopathy (DA)

Twenty-five placentas (40%) showed evidence of DA, including 17 of 33 with measured thrombin generation. Women who later developed placental markers of DA had higher prepregnancy systolic blood pressure than those without DA (118 ± 10 vs 114 ± 11 mmHg; p=0.02) and lower prepregnancy plasma volume corrected for lean body mass (PV/LBM), 60.0 ± 7.3 mL/kg compared to mean 66.7 ± 7.5 mL/kg, p= 0.0001, in those without DA. In early pregnancy, women subsequently identified with DA had decreased uterine blood flow, 277 ± 144 compared to 401 ± 221 mL/min, p=0.046, for placentas without DA. Differences in maternal physiology and TG were also detected in late pregnancy. In the third trimester, systolic blood pressures were significantly elevated in women with DA (107 ± 12 vs. 100 ± 9 mmHg; p=0.02), and plasma volume corrected for lean body mass remained lower than for women without DA (92 ± 17 vs. 101 ± 12 mL/kg; p=0.05). In the TF+TM condition of the TG assays, the maximum rate of thrombin generation was higher in those with subsequent classification of a placenta with DA (maximum rate 145 ± 58 vs. 104 ± 35 nM/min; p=0.03). Thrombin generation parameters from TF+TM assay conditions for all reported placental MVM criteria and study visits are presented in Table 3. No differences between parameters from the TF-only assay condition identified for any MVM.

### 3.3. Infarcts and Agglutination (INFAGG)

Twenty-eight placentas (46%) showed evidence of infarcts and/or agglutination, including 16 with thrombin generation. There were no differences in prepregnancy characteristics identified in comparisons of women who did and did not develop placental evidence of INFAGG. In the first trimester of pregnancy, women who later developed INFAGG had more compliant popliteal arteries, with decreased beta stiffness index (13.0 ± 7.1 vs 25.1 ± 22.7, p=0.02) and increased vessel distensibility (0.0012 ± 0.0008 vs 0.0008 ± 0.0006 10^−3^*mmHg^−1^ p=0.07) compared to those without INFAGG. TG was increased in TF-initiated TG in the presence of thrombomodulin (mean peak 291 ± 324 vs. 237 ± 83 nM p=0.046; mean EPT 1541 ± 326 vs 1250 ± 451 nM*min 0.04). There were no differences in thrombin generation in late pregnancy.

### 3.4. Accelerated Villous Maturation (AVM)

Twenty placentas (32%) showed evidence of AVM, including 7 of 33 with measured thrombin generation data. There were no differences detected prior to pregnancy between women who did or did not develop AVM. In the first trimester, those who were subsequently characterized as having AVM had increased uterine pulsatility (1.8 ± 0.03 vs 1.4 ± 0.04; p< 0.0001) and resistance indices (0.76 ± 0.05 vs 0.68 ± 0.08; p< 0.0001) and decreased uterine blood flow (294 ± 121 vs 397 ± 229 mL/min; p=0.38). Popliteal artery distensibility was also lower (0.0007 ±0.0004 vs 0.001 ± 0.0008 10^−3*^mmHg^−1^, p=0.38). TG in the first trimester in the presence of thrombomodulin was also increased in women who developed AVM (peak 327 ±6 0 vs 248 ± 74 nM, p=0.01; rate 147 ± 39 vs 105 ± 34 nM/min, p= 0.008; ETP 1711 ± 251 vs. 1316 ± 410 nM*min, p=0.02) in women who developed AVM. In the third trimester there were no significant differences in TG profiles by villous maturation status. Uterine hemodynamics and popliteal distensibility were not assessed at this visit

### 3.5. Distal Villous Hypoplasia (DVH)

Eleven placentas (17%) showed evidence of distal villous hypoplasia, including six of the 33 with measured thrombin generation data. Women who would later develop placental evidence of DVH had lower plasma volume corrected for lean body mass (PV/LBM 58.3 ± 9.8 vs. 65.3 ± 7.2 mL/kg; p=0.008), and lower arterial distensibility (0.00076 ± 0.00039 vs 0.00108 ± 0.00078 10^−3^*mmHg^−1^; p=0.002) in early pregnancy compared to those without DVH. In early pregnancy, PV/LBM remained lower (63.3 ± 10.7 vs. 75.6 ± 9.2 mL/kg; p=0.002), and maternal blood pressure was higher (systolic 121 ± 12 vs. 113 ± 11 mmHg, p= 0.04; diastolic 70 ± 9 vs 64 ± 7 mmHg, p= 0.04; mean arterial pressure 89 ± 10 vs. 82 ± 8 mmHg, 0.02) in women with placental evidence of DVH compared to those without. There were no physiologic differences in late pregnancy, or in thrombin generation at any time point.

### 3.6. Placental Hypoplasia

Eighteen placentas (29%) had a placental weight below the 10^th^ percentile when adjusted for gestational age by both Naeye [25] and Pinar et al [24]. Increasing placental percentile was associated with increased pulse measured prepregnancy (r=0.27, p=0.03), in early pregnancy (r=0.30, p=0.03), and in late pregnancy (r=0.30, p=0.02). In early pregnancy, increased TF+TM thrombin generation parameters were associated with decreased placental weight percentile (peak r= −0.34, p=0.05; ETP r= −0.44, p=0.009). Placental weight percentile was not associated with TG prior to pregnancy or in the third trimester.

### 3.8. MVM Composite Score

Eighteen placentas (31%) showed evidence of two or more of the above MVM characteristics. Prepregnancy, the odds of having 2 or more features of MVM was increased with increased response to volume challenge, an index of reduced vascular compliance, (AUC cardiac output odds ratio = 1.08 L/min, 95% CI: 1.01 to 1.2) and decreased pulse (OR= 0.92, 95% CI: 0.86 to 0.99). In the first trimester, the odds of having 2 or more features of MVM increased with decreasing plasma volume expansion from prepregnancy levels (OR= 0.92, 95% CI: 0.85 to 0.999). Late pregnancy plasma volume expansion was similarly predictive of the presence of 2 or more features of MVM (OR 0.95, 95% CI: 0.90 to 0.996). We did not see an independent relationship of the presence or absence of preeclampsia with placental MVM in this cohort.

## 4. Discussion

While the etiology of overall MVM is frequently identified as a result of poor implantation and placental development, the underlying reasons for this unifying characterization likely differ. As we demonstrate, assessment of longitudinal cardiovascular health and thrombin generation potential point to differing origins for distinct MVM lesions. Women who later developed placentas with DA had prepregnancy profiles consistent with an altered cardiovascular phenotype, suggesting these placental lesions may originate in response to underlying maternal physiology. Prepregnancy maternal characteristics did not differ based on placental evidence of infarcts and agglutination or accelerated villous maturation. However, adaptation to pregnancy, particularly in measures of cardiovascular adaptation within early pregnancy, were significantly impaired in women who developed these lesions. A similar impaired adaptation to pregnancy of the uterine vasculature was identified in DA, suggesting that the many features classifying DA may themselves have differing etiologies. Thrombin generation profiles similarly differed by MVM lesion type, with the development of accelerated villous maturation and infarcts and agglutination occurring with increased thrombin potential in early pregnancy, while decidual arteriopathy was associated with increased thrombin generation in late pregnancy. The variation of differing placental features and the time points in which differences are identifiable may point to a developmental timeline for the origins of the identified histopathologic lesions, as well as to potential etiologies of these lesions.

Maternal adaptation to pregnancy sets the stage for pregnancy outcomes, and a woman’s prepregnancy health in turn influences the maternal ability to adequately adapt to pregnancy. Our findings suggest that some placental pathologies, including decidual arteriopathy may be influenced by underlying prepregnancy status, which likely influences the maternal physiologic adaptation around the time of implantation. While the mechanisms have not been fully elucidated, insufficient remodeling of the spiral arteries results in inadequate blood low, leading to uteroplacental hypoxia, dysregulation maternal-fetal nutrition, and increased inflammation [1, 9, 10]. The origin of many placental lesions is not fully understood, DA is thought to originate in early gestation, and is composed of several features, including acute atherosis, fibrinoid necrosis, mural hypertrophy, chronic perivasculitis, absence of spiral artery remodeling, arterial thrombosis, or persistence of intramural endovascular trophoblast in the membrane roll, basal plate, or both [6, 16]. Early pregnancy uterine artery remodeling is thought to be the primary influence on ultimate placental development; however, recent discoveries have identified that defective placentation may be the primary cause of poor arterial adaptation [26].

Pregnancies impacted by preterm or severe preeclampsia have the highest frequency of having features of MVM, most frequently AVM, DVH, DA, and infarcts when compared to either term preeclampsia or normotensive pregnancies [7, 27]. Placental evaluation from preterm and term preeclampsia patients similarly demonstrate that severe preeclampsia is more frequently associated with placental abnormalities [28]. Stark et al examined the frequency of histologic features of MVM with birthweight and placental outcomes. They reported increased incidence of syncytial knots, DVH, villous agglutination and infarcts with early onset preeclampsia, increased placenta site giant cells with decreased birth weight, and increased birthweight with decreased syncytial knots [29]. These findings are consistent with the concept that features of MVM have different etiologies, and thus the pathologic process for MVM lesions associated with term and preterm preeclampsia differ. As our sample size was small, we did not have the power to detect differences in MVM lesions according to preeclampsia onset. We did not attempt to reproduce the finding that placentas from female infants in cases of early preeclampsia have increased frequencies of DA than male fetuses in early onset preeclampsia due to our small volume of cases [30]. Despite the associations with disease, Romero et al reported 35.7% of 944 normal pregnancies also had placental evidence of MVM [8]. The frequency of placentas with lesions observed, at 70% overall and particularly for DA, were higher than anticipated. This may be due to the study population, which specifically recruited women with a history of preterm preeclampsia and nulliparous women, both groups with increased risk of preeclampsia and other adverse pregnancy outcomes. Overall, within our cohort we did not see a specific association of preeclampsia, either preterm or term with the placental MVM lesion reported.

The importance of maternal prepregnancy health status on pregnancy outcomes is widely accepted. However, the concept of subclinical predictive phenotypes that contribute to poor outcomes and increase later disease risk are relatively novel. Our lab has studied how prepregnancy cardiovascular health and adaptation to pregnancy impact pregnancy outcomes, particularly in the development of subsequent preeclampsia. Prior studies have identified the relationship between prepregnancy uterine artery resistance [11, 12], and subclinical peripheral vascular phenotypes [13, 14], with distinct pathophysiologic pregnancy adaptations. Ongoing research demonstrates that prepregnancy characteristics influence adaptation to pregnancy, and thus are in part responsible for pregnancy outcomes and longer term cardiovascular and metabolic risk. Increased prepregnancy blood pressure and a smaller decline in diastolic blood pressure have also been reported to occur in women who develop evidence of MVM, after adjustment for covariates [15]. This finding suggests a relationship between MVM and unique vascular adaptations to pregnancy, potentially including prepregnancy physiology. In support of this placental development, particularly with evidence of MVM, may be related to future risk of cardiovascular disease in these women [16]. Placental development, particularly evidence of MVM, is an early indication of subclinical deficiencies in the maternal hemodynamic state, which may be related to future risk in these women [16]. Women who had been diagnosed with preeclampsia, with and without decidual vasculopathy were evaluated 7 months postpartum, and those with DA were found to have increased diastolic blood pressure, lower left ventricular stroke volume, higher total peripheral vascular resistance, and lower plasma volume compared to those without DA [31]. Women who had characteristics of MVM identified during placental pathology had poor cardiovascular profiles, including higher blood pressure and atherogenic lipids more than a decade after delivery [32]. Additionally, if lesions occurred in a prior pregnancy, there is an increased likelihood of reoccurrence [33]. The inclusion of both prior preterm preeclamptics and nulliparous study subjects may explain our observed high frequency of any MVM lesion.

Coagulation markers during early pregnancy and the associated increased risk of venous thromboembolism during the pregnant state demonstrate a changing equilibrium between the need for enhanced blood flow and angiogenesis required for placental development and continuous clotting and lysing which allow for these adaptations to occur. Pregnancy is recognized as a hypercoagulable state, resulting in elevated risk of venous thromboembolism through upregulation of estrogen levels, increases in clotting factors and tissue factor, and a decrease in fibrinolytic activity [34]. The extrinsic pathway of the coagulation cascade is initiated by TF, leading to thrombin generation and results in fibrin clot formation. *In vitro* thrombin generation assays capture the individual’s potential for thrombin generation, allowing for better understanding of the individual coagulation profiles than assessments of individual factor levels. Thrombin generation has a wide degree of individual variability, though within-individual, levels are typically stable over time in healthy individuals [35–37]. Many studies assess plasma levels of coagulation factors, including D-dimer, PAI-1 and TPA with pregnancy advancement are widely accepted [34, 38]. Fewer studies have longitudinally assessed thrombin across pregnancy, though McLean et al. reports a significant increase in tissue-factor initiated thrombin generation from prepregnancy through late pregnancy [39]. Our results are consistent with the increase in thrombin generation through pregnancy. Additionally, our results suggest that hyper-procoagulant thrombin potential changes observed as early as the end of the first trimester may result in placental lesion formations.

Women with preeclampsia have been shown to have increased thrombin generation when compared to women with small for gestational age infants and healthy outcomes [18]. Placental pathology of these pregnancies found that women with evidence of DVH was associated with increased maternal tissue factor compared to those without this pathological finding [18]. This was not evaluated in our study.

The main limitation of this study results from its size, however we are unaware of studies that have so thoroughly assessed maternal health from the prepregnancy state through delivery. The assessment of individual characteristics of MVM in relation to prepregnancy maternal health, changes within uterine hemodynamics and the cardiovascular system as adaptation to pregnancy, and thrombin generation profiles throughout pregnancy are novel. These assessments allow for potential identification of developmental timelines for the origins of distinct placental development and allow for determination of whether these factors may originate from underlying maternal factors, from maladaptation to early placental development, or originate later in pregnancy.

## Figures and Tables

**Table 1.** Prepregnancy demographics, birth outcomes, and frequency of placental evidence of MVM lesions.

Characteristic	Descriptor (n=63)
**Baseline Characteristics**	
Age, years	31 ± 4
BMI, kg/m^2^	24 ± 5
Race, % Caucasian	(54) 86%
History of Preeclampsia	(12) 20%
**Delivery Outcomes**	
Gestational Age, days	276 ± 12
Infant Sex, male	(37) 59%
Birth Weight, grams	3354 ± 6570
Birth Weight Percentile	49 ± 27%
Preeclampsia	(9) 14%
**Placental Characteristics**	
Placental Mass, grams	472 ± 98
Placental Mass Percentile	36 ± 31%
Birthweight/Placental Weight Ratio	7.2 ± 1.1
**Any MVM lesion**	(53) 70%
Decidual Arteriopathy	(25) 40%
Infarcts and Agglutination	(28) 44%
Accelerated Villous Maturation	(20) 32%
Distal Villous Hypoplasia	(11) 17%
Placental Hypoplasia	(18) 29%
**Number of MVM lesions**	
None	(10) 16%
One	(15) 24%
Two	(22) 35%
Three	(13) 21%
Four	(3) 4%

Data is presented as mean

± standard deviation or (n) frequency

**Table 2.** Physiologic assessments of the study population, means at each study visit.

Physiologic Characteristics of Participants	Prepregnancy (n= 63)	First Trimester (n= 54)	Third Trimester (n= 57)
Cycle Day or Gestational Age, days	9 ± 4	90 ± 6	216 ± 5
Lean Body Mass (LBM), %	40 ± 5	‐	‐
Fat Body Mass, %	24 ± 11	‐	‐
Insulin Resistance, HOMA-IR	1.09 ± 0.82	‐	‐
Plasma Volume/LBM, mL/kg	64.0 ± 8.0	72.7 ± 10.2	97.8 ± 13.8
Plasma Volume Increase from Prepregnancy, %	‐	14 ± 16	54 ± 22
Systolic Blood Pressure, mmHg	118 ± 10	114 ± 11	114 ± 10
Diastolic Blood Pressure, mmHg	68 ± 6	65 ± 8	63 ± 9
Mean Arterial Pressure, mmHg	88 ± 7	83 ± 9	81 ± 8
Pulse, bpm	63 ± 9	67 ± 10	78 ± 10
Brachial Pulse Wave Velocity, m/sec	7.88 ± 1.52	7.82 ± 1.79	7.20 ± 1.43
Popliteal Pulse Wave Velocity, m/sec	3.78 ± 0.45	3.83 ± 0.43	3.65 ± 0.48
Beta Stiffness Index	18.3 ± 19.3	19.6 ± 18.3	‐
Distensibility, 10^−3^ mmHg^−1^	0.0010 ± 0.0007	0.00098 ± 0.00072	‐
Response to Volume Loading, mmHg	104 ± 111	‐	‐
Cardiac Output (C), L/min	4.6 ± 1.1	5.1 ± 1.0	6.0 ± 1.1
Uterine Blood Flow, mL/min	39.6 ± 20.2	363.9 ± 204.9	‐
Uterine Pulsatility Index	3.18 ± 1.24	1.51 ± 0.41	‐
Uterine Resistance Index	0.91 ± 0.07	0.70 ± 0.08	‐
Uterine Index (UBF/CO), %	0.89 ± 0.51	7.66 ± 5.22	‐

Data are presented as mean

± standard deviation unless otherwise indicated.

‐ indicates that the assessment was not completed during that study visit.

**Table 3.** Results of the 33 placentas with thrombin generation assay results in the presence of tissue-factor initiation with and without the presence of thrombomodulin.

Visit	Placental MVM	MVM?	TF-Initiated Thrombin Generation Peak, nM	TF-Initiated Thrombin Generation ETP, nM*min	TF+TM Thrombin GenerationPeak, nM	TF+TM Thrombin Generation ETP, nM*min
Prepregnancy	Decidual Arteriopathy	Yes, n= 17 No, n= 16	259 ± 43 262 ± 41	1606 ± 399 1540 ± 250	122 ± 47 141 ± 46	593 ± 250 683 ± 246
	Infarcts and Agglutination	Yes, n= 16 No, n= 17	263 ± 36 259 ± 46	1559 ± 226 1585 ± 407	138 ± 37 126 ± 54	668 ± 189 613 ± 297
	Accelerated Villous Maturation	Yes, n= 7 No, n= 26	276 ± 42 260 ± 40	1626 ± 170 1557 ± 359	143 ± 39 129 ± 49	719 ± 252 618 ± 247
	Distal Villous Hypoplasia	Yes, n= 6 No, n= 27	284 ± 54 256 ± 37	1773 ± 572 1527 ± 239	142 ± 43 129 ± 48	719 ± 291 622 ± 240
Early Pregnancy	Decidual Arteriopathy	No, n= 17 No, n= 16	383 ± 50 369 ± 55	2299 ± 355 2220 ± 322	273 ± 64 258 ± 90	1453 ± 338 1349 ± 477
	Infarcts and Agglutination	Yes, n= 16 No, n= 17	390 ± 57 361 ± 44	2297 ± 349 2218 ± 326	291 ± 64* 237 ± 83	1541 ± 326* 1250 ± 451
	Accelerated Villous Maturation	Yes, n= 7 No, n= 26	409 ± 77 367 ± 41	2368 ± 343 229 ± 334	327 ± 60* 248 ± 74	1712 ± 251* 1316 ± 410
	Distal Villous Hypoplasia	Yes, n= 6 No, n= 27	377 ± 54 376 ± 53	2263 ± 361 2258 ± 337	269 ± 67 264 ± 80	1401 ± 339 1400 ± 430
Late Pregnancy	Decidual Arteriopathy	Yes, n= 17 No, n= 16	384 ± 345 349 ± 41	2206 ± 324 2134 ± 215	308 ± 94* 247 ± 70	1503 ± 491 1262 ± 348
	Infarcts and Agglutination	Yes, n= 16 No, n= 17	370 ± 48 361 ± 73	2215 ± 269 2125 ± 275	286 ± 65 268 ± 105	1468 ± 355 1296 ± 493
	Accelerated Villous Maturation	Yes, n= 7 No, n= 26	377 ± 62 362 ± 32	2227 ± 177 2153 ± 293	311 ± 65 268 ± 91	1525 ± 195 1340 ± 474
	Distal Villous Hypoplasia	Yes, n= 6 No, n= 27	371 ± 74 364 ± 60	2147 ± 229 2173 ± 284	276 ± 122 277 ± 81	1290 ± 527 1399 ± 420

Data presented as mean

± standard deviation with

* representing statistical significance between the presence/absence of individual placental characteristics of MVM.

## Data Availability

Data is available by request. Please contact the corresponding author.
